# Person centered prediction of survival in population based screening program by an intelligent clinical decision support system

**Published:** 2017

**Authors:** Reza Safdari, Elham Maserat, Hamid Asadzadeh Aghdaei, Amir hossein Javan Amoli, Hamid Mohaghegh Shalmani

**Affiliations:** 1 *Allied Medical Sciences School, Tehran University of Medical Sciences, Tehran, Iran *; 2 *School of Management and Medical informatics, Tabriz University of Medical Sciences, Tabriz, Iran*; 3 *Basic and Molecular Epidemiology of Gastrointestinal Disorders Research Center, Research Institute for Gastroenterology and Liver Diseases, Tehran, Iran*; 4 *Islamic Azad University*; 5 *Gastroenterology and Liver Diseases Research Center, Research Institute for Gastroenterology and Liver Diseases, Shahid Beheshti University of Medical Science, Tehran, Iran*

**Keywords:** Colorectal Cancer, Survival rate, Decision support system, Screening

## Abstract

**Aim:**

To survey person centered survival rate in population based screening program by an intelligent clinical decision support system.

**Background:**

Colorectal cancer is the most common malignancy and major cause of morbidity and mortality throughout the world. Colorectal cancer is the sixth leading cause of cancer death in Iran. In this survey, we used cosine similarity as data mining technique and intelligent system for estimating survival of at risk groups in the screening plan.

**Methods:**

In the first step, we determined minimum data set (MDS). MDS was approved by experts and reviewing literatures. In the second step, MDS were coded by python language and matched with cosine similarity formula. Finally, survival rate by percent was illustrated in the user interface of national intelligent system. The national intelligent system was designed in PyCharm environment.

**Results:**

Main data elements of intelligent system consist demographic information, age, referral type, risk group, recommendation and survival rate. Minimum data set related to survival comprise of clinical status, past medical history and socio-demographic information. Information of the covered population as a comprehensive database was connected to intelligent system and survival rate estimated for each patient. Mean range of survival of HNPCC patients and FAP patients were respectively 77.7% and 75.1%. Also, the mean range of the survival rate and other calculations have changed with the entry of new patients in the CRC registry by real-time.

**Conclusion:**

National intelligent system monitors the entire of risk group and reports survival rates by electronic guidelines and data mining technique and also operates according to the clinical process. This web base software has a critical role in the estimation survival rate in order to health care planning.

## Introduction

Colorectal cancer (CRC) is a major cause of morbidity and mortality throughout the world (1, 2), with an annual incidence of 1 million cases (3, 4, 5) and an annual mortality of more than 500,000 cases. CRC is the second most common cause of cancer mortality (6) and one of most malignancies cancers in Iran (7,8). Recent epidemiological studies were demonstrated the increasing incidence trend of CRC in Iran (9,10). According to consensus, there are more than 3641 new cases of CRC diagnosed in Iran (11). Colorectal cancer is the sixth leading cause of cancer death and annually there are around 2262 deaths from CRC in Iran (11, 12). The overall 5-year survival rate for colorectal cancer patients was 61% (13, 14). Annual incidence of CRC has increased over the next two decades in developing countries (15, 16, 17, 18). Socioeconomic status such as race, ethnicity, geographical and economic characteristics has a significant role on CRC incidence rates, mortality rates, and survival rates (19, 20). More studies are demonstrated that colorectal cancer incidence and mortality are reduced with regular screening (21).

Decision Support System (DSS) as a computer-based system improves preventive care services (22) and extracts knowledge from routine care data. Person centered prediction of survival is one of this knowledge (23). Machine-learning services of DSS accurately predict outcomes in CRC. Also health care providers can be used of the DSS tools to facilitate treatment and screening planning (24). Computer-aided decision support systems improve quality of care by providing of the patient specific alert, reminders and recommendations (25, 26). CDSS play critical role in clinical setting by several artificial intelligence methods and techniques (27). Cosine similarity is one the clustering technique (28). Cosine similarity estimate degree of similarity between two items or users. Cosine similarity as a data mining method can be used for measuring similarity rate between two data objects. Similarity is detected distances between features of the objects. High degree of similarity and low degree of similarity was defined by distance interval. All of the features values must be normalized because even one feature is changed dominating the distance calculation (29). Cosine similarity was introduced as the decision rule for query detection in large data sets (30).

 In this survey, we used cosine similarity as data mining technique for estimating survival of at risk groups in the screening plan. High volume, high velocity, and high variety information products in the CRC screening plan. However, intelligent approaches as innovation strategy facilitate clustering of data and decision making process. Decisions of this system can be useful for understanding the screening modalities, survival rate of cancer and recommended time intervals of screening. The aim of this study is to predict the survival in the population based screening program by an intelligent clinical decision support system.

## Materials and Methods

In the first step, we defined minimum data set (MDS). MDS was approved by experts and reviewing literature. Vital status of the covered population in CRC registry was established by matching with medical record and telephone enquiries. In the second step, MDS were coded by python language and matched with cosine similarity formula. Also MDS such as BMI (Body Mass Index) was calculated by coding method in intelligent system. Sample of coding: 

 #Calculate BMI

 if row['Weight'] and row['Height'] and row['Weight'] != 0 and row['Height'] != 0:

 bmi = row['Weight']/(row['Height']/100)**2

 else:

 bmi = np.nan

 if bmi < 18.5:

 bmi_list.append(1)

 elif bmi < 24.9:

 bmi_list.append(2)

 elif bmi < 29.9:

 bmi_list.append(3)

 else:

 bmi_list.append(4)

 else:

 persian_bdate = persian_bdate.split("-")

 gregorian_bdate = jdate.jd_to_gregorian(

 jdate.persian_to_jd(int(persian_bdate[0]), int(persian_bdate[1]), int(persian_bdate[2])))

 age = int(time.strftime("%Y")) - gregorian_bdate[0]

 if age < 45 :

 age_list.append(1)

 elif age < 65:

 age_list.append(2)

 else:

 age_list.append(3)

Cosine similarity implementation in python:

#!/usr/bin/env python

from math import*

def square_rooted(x):

 return round(sqrt(sum([a*a for a in x])),3)

def cosine_similarity(x,y):

 numerator = sum(a*b for a,b in zip(x,y))

 denominator = square_rooted(x)*square_rooted(y)

 return round(numerator/float(denominator),3)

print cosine_similarity([3, 45, 7, 2], [2, 54, 13, 15])

Finally, survival rate by percent was illustrated in user interface of intelligent system. Intelligent system was designed in PyCharm environment. PyCharm is an integrated development dnvironment (IDE) applied for programming in Python. This system has rule base environment. These rules were designed by risk assessment guidelines. Survival rate and risk group of covered population synchronously were presented in the user interface.

## Results

**Figure 1:**
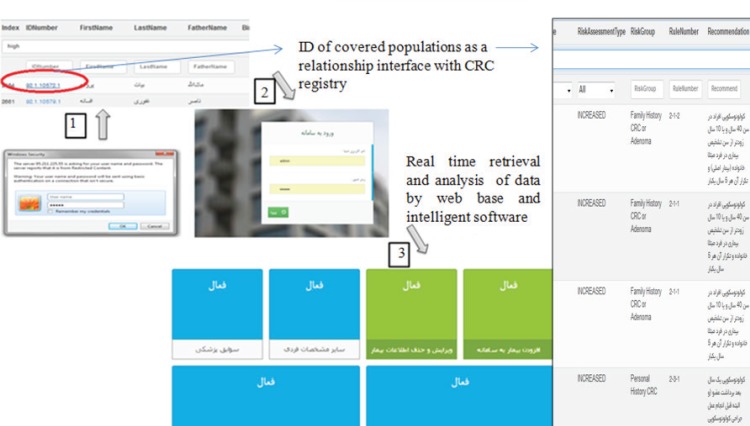
Process of the estimated survival rate for covered population in a screening plan

**Figure 2:**
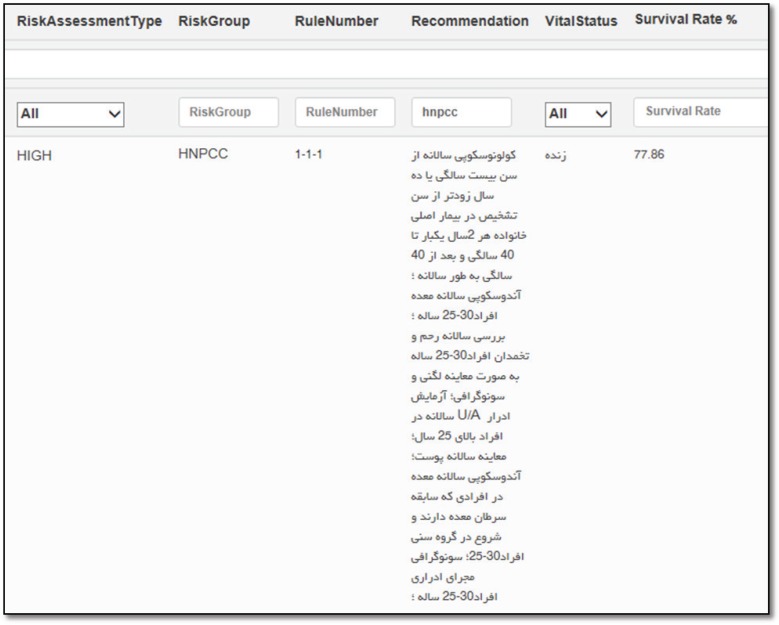
An estimated survival rate for HNPCC patients

Minimum data set related to survival comprise of clinical status, past medical history, life style and socio-demographic information. MDS related to survival of CRC was illustrated in table 1. Main data elements of intelligent system consist demographic information, age, referral type, risk group, recommendation and survival rate. This system estimates survival rate of alive patients by dead patients. Estimation was conducted by similarity. Intelligent system integrates with CRC registry. Information of covered population as a comprehensive database was connected to intelligent system and survival rate estimated for each patient (figure 1). Also expert system report person-specific advice about risk assessment and survival status. Mean range of survival of HNPCC patients were 77.7%. One of the HNPCC sample was shown in figure 2. HNPCC is subgroup of high risk group. The high risk group comprises of 8 subgroups (FAP, AFAP, Suspected FAP, Suspected FAP, HNPCC, Suspected HNPCC, MYH, IBD). Generally, survival rate of high risk group was low. Mean range of survival of FAP patients were 75.1 %(figure 2). Mean range of survival rate and other calculation change with the entry of new patient in CRC registry. Intelligent clinical decision support system enables to update all of information and consensus in real time. As an example of real time changes, we evaluated system by edition of a suspected HNPCC patient. An individual should meet Amsterdam II criteria and then Bethesda II guideline as a suspected HNPCC patient. If a suspected HNPCC patient have a mutation that is identified by molecular genetic testing, intelligent clinical changes a suspected HNPCC patient status to HNPCC patient by result of genetic testing. Patient with abnormal IHC and high MSI was introducing as a HNPCC patient by guidelines of expert system. 

This system is web base software and has two method search, including global search and special search. Special search include search by ID number, screening recommendation (Screening method, age of screening, interval screening), risk and survival status.

## Discussion

Studies have shown, CRC is one of the significant cancer and is responsible for high mortality in the worldwide (31,32). However, analyzing of survival rate of CRC is highly important field. Different survival rates of patients with colorectal cancer have been surveyed in several studies (33,34,35,36,37). Intelligent software of screening plan was evaluated survival rate of first degree relatives of CRC patients and CRC probands. We defined MDS for accurate calculation of survival rate. Quality improving cancer patient care will take place only through the systematic collection of MDS and use of accurate data elements (38). Also, comparing the collected data from different studies was conducted by standardization and uniformity of data elements (39). In this survey, minimum set of data elements agreed for standard reporting at a national level. We identified core dataset for intelligent system that assist to predict many variables such as vital status of first and second relatives of patients in the future.

Stigliano, et al. (37) reported improved prognosis of cancer in patients with HNPCC with a 5 year cumulative survival (94.2%) versus sporadic CRC (75.3%). In this survey, mean range of survival of HNPCC patients was 77.7%. This survival rate is lower rather than to other countries. Survival rate estimation of cancer play key role for health care planning. Survival rate reports can be used for researching base on cancer prevention (40, 41). Reliable research presents the complex mechanisms and pathways in the tumorigenesis and natural history of HNPCC tumors and there aren’t clinical indicators of good prognosis in HNPCC-related colorectal carcinomas. Thus, CRC screening guidelines must be based on the improved expected survival rates of all patients. Also, the mean range of survival of FAP patients were 75.1%. Studies has been shown screening of families with FAP can reduce CRC mortality (42). Recent studies have been shown multidimensional role of guidelines for preventive care services (21). Computer-aided consultation improves real time management (43). Screening guidelines should be integrated with the care process in order to providing suitable patient-specific advices. Also, national intelligent system monitors the entire risk group and report survival rates by electronic guidelines and data mining technique and operates according to clinical process. This web base software has a critical role in optimal interactions between colonoscopy, pathology and laboratory data. 

After more than a decade of development of numerous computerized systems, studies on the most effective implementation of intelligent risk assessment systems and guideline base system is still lacking. A big obstacle for implementing of DSS is the difficulty in obtaining accurate and complete data required for decision making. Hence uniform templates for data collecting and standard reporting are essential. Quantitative and qualitative evaluation of data can be assisted to precise estimate of the survival rate. 

In conclusion, low survival rate of HNPCC and FAP indicates the extremely urgent needs for health authorities to agreed measures of CRC screening. Early detection and counting of patient care improve expected survival rate of CRC. Identifying an asymptomatic person at risk and needing diagnosis and follow-up treatment could lead to increasing of survival rate.

Also, it is recommended to apply intelligent system for improving quality of care and reduce cost. Decisions of computer base system could be a suitable tool to assist clinicians understanding cancer risk, the screening approach, survival rate, recommended screening time intervals and preferences.

**Table 1:** Minimum Data Sets related to survival of CRC

**Core Data Element**	**Variable**	**Variable Value**
**Socio-demographic Data**	Current age and diagnosis age	"45 > " = 1 / "65-45" = 2 /"65 < " = 3
	Sex	Female =1/ Male = 2/ Transsexual = 3
	Education level	Illiterate = 1 / Primary school = 2 / High school = 3 / University = 4
	Marital Status	Married = 1 / Single = 2 / Divorced = 3 / Widowed = 4
	Ethnicity	Fars = 1 / Turk = 2 / Kord = 3 / Lor = 4 / Other =1
	Religion	Muslim =1/ Christian=2/ jewish=3/ Zoroastruan=4/ Other=5
	Location	City=1/Village =2
**Life-style**	BMI	18.5>=1/18.5-24.9=2/25-29.9=3/30<=4
	Exposure to chemical weapons and pollutant-material	Yes=1/NO=0/Unknown=3
	Tobacco Use	Yes=1/NO=0/Unknown=3
	Alcohol Use	Yes=1/NO=0/Unknown=3
	Oral narcotic substance Use	Yes=1/NO=0/Unknown=3
	IV Drug User	Yes=1/NO=0/Unknown=3
	NSAIDs Use	Yes=1/NO=0/Unknown=3
**Tumor Classification**	Tumor size (grad I,II)	20mm> =1/20mm<=2; 35mm> =1/35mm<=2
	Tumor topology	Cecum =1, Ascending colon=2, Hepatic flexure=3/ Transverse colon=4/Splenic flexure=4/Descending colon=5/Sigmoid colon=6/ sigmoid colon=7/Colon=8/ rectosigmoid=9/ junctionRectum=10
	Tumor Morphology	Adenocarcinoma=1/ Mucinous adenocarcinoma=2/ Signet Ring Cell Carcinoma=3/ Squamous cell carcinoma=4/ Squamous-cell adenocarcinoma=5/ medullary carcinoma=6/
	Histological Differentiation Grade	Grade cannot be assessed (undetermined grade) =1/Well differentiated (low grade) =2/ Moderately differentiated (intermediate grade) =3/ Poorly differentiated (high grade)=4/Undifferentiated (high grade)=5
	Pt grade	0 = T0/ T1=1/T2=2/ T3=3/ T4=4
	Pn grade	N0=0/N1=1/ N2=2
	Pm grade	M0=0/M1=1
	Pathologic grade	I=1/II=2/III=3/IV=4
	Advancing grade	Primary grade=1/Advanced grade=2
	Treatment	Surgery=1/chemotherapy, Radiotherapy =2/Biopsy=3
	HNPCC	Yes=1/No=0
	FAP	Yes=1/No=0
**Past Medical History**	Diabetes Mellitus	Yes=1/No=0/Unknown=3
	Hypertension disorder	Yes=1/No=0/Unknown=3
	IBD	Yes=1/No=0/Unknown=3
	Personal history of CRC	Yes=1/No=0/Unknown=3
	Family history of CRC	Yes=1/No=0/Unknown=3
**Symptoms**	Abdominal perforation	Yes=1/No=0
	FUO	Yes=1/No=0
	Obstructive Symptoms	Yes=1/No=0
	Abdominal Pain	Yes=1/No=0
	Anemia	Yes=1/No=0
	Rectal Bleeding	Yes=1/No=0
	Change in Bowel Habit	Yes=1/No=0
	Weakness	Yes=1/No=0
	Weight Loss	Yes=1/No=0
**Vital Quality**	Survival quality	Normal activity=1/Non normal activity=2/Dead=3
	Vital status	Alive=1/Dead=0
	Survival time	By month
